# Heterogeneity in multistage carcinogenesis and mixture modeling

**DOI:** 10.1186/1742-4682-5-13

**Published:** 2008-07-21

**Authors:** Sandro Gsteiger, Stephan Morgenthaler

**Affiliations:** 1 Institute of Mathematics, Swiss Federal Institute of Technology, Lausanne, Switzerland

## Abstract

Carcinogenesis is commonly described as a multistage process, in which stem cells are transformed into cancer cells via a series of mutations. In this article, we consider extensions of the multistage carcinogenesis model by mixture modeling. This approach allows us to describe population heterogeneity in a biologically meaningful way. We focus on finite mixture models, for which we prove identifiability. These models are applied to human lung cancer data from several birth cohorts. Maximum likelihood estimation does not perform well in this application due to the heavy censoring in our data. We thus use analytic graduation instead. Very good fits are achieved for models that combine a small high risk group with a large group that is quasi immune.

## Introduction

Cancers can arise in virtually any part of the body, and although there are many tissue specific properties, a general multistage framework for carcinogenesis holds for most cancer types. More precisely, cells must undergo an evolutionary process involving several stages and leading finally to a cell that has completely lost proliferation control. In a first step, called initiation, mutations transform stem cells into intermediate states. Such initiated cells may give rise to pre-neoplastic lesions via accelerated growth. Eventually, a cell out of such a clone may experience further mutations and be transformed into a malignant tumor cell. This second step comprising clonal expansion and final malignant transformation is commonly called promotion. This multistage scheme shows the inherently random aspect of carcinogenesis: mutations happen at random times and stochastic growth processes are involved.

Mathematical models of carcinogenesis have been studied for about fifty years. Some of the earliest attempts to build biologically based quantitative descriptions are [1] and [2], who explained cancer as the result of a sequence of mutations. A widely accepted model was proposed in [3] and [4]. Their two-stage clonal expansion (TSCE) model was explicitly formulated in terms of an initiation stage and a promotion stage. This approach stressed the importance of both mutations and clonal expansion in the process leading to cancer. The TSCE model has found many applications and extensions. One example is the multistage model, which takes up the same structure but allows for more than two stages. Due to this long and evolving story, we should not have in mind a single model when talking about the multistage model. We should rather have in mind a cascade of nested models that starts from a fundamental idea and incorporates through its evolution more and more biological detail. Excellent reviews of stochastic carcinogenesis modeling can be found in [5] and [6].

One part of the recent extensions tries to take population heterogeneity into account. Such heterogeneity can result from sources such as genetic variation, exposure to carcinogens due to either changes in environment or occupation, and differences in lifestyle (the most prominent factors being smoking and diet). In [7] a mixture of a one stage model and a two stage model was used to describe the heritable and the sporadic form of Retinoblastoma, a cancer of the eye caused by mutations in a single tumor suppressor gene. Other approaches incorporate heterogeneity via standard frailty modeling, where the common baseline hazard *h*_0_(*t*) is multiplied by a non-negative random variable *Z *in order to model the individual hazard *h*_*ind*_(*t*) = *Zh*_0_(*t*), see for example [8], and [9] for such an approach.

In this text, we take up the work by [10]. These authors introduce two new population parameters to describe heterogeneity. The first one, called the fraction at risk *F*, is used to distinguish between susceptibles and a postulated group of immune individuals. The second one, called the fraction of deaths due to cancer among all deaths due to either cancer or related competing causes *f*, models competing related risks. They fit their model to US lung cancer incidence data from several birth cohorts. The parameters *F *and *f *present an abstract way describe population heterogeneity and are not linked to a specific biological process. Therefore, the above mentioned authors state that other modeling strategies could be tested. The present work gives such an attempt. We take up the same multistage model, but we will use mixture models to allow for variability among individuals. This allows us to introduce heterogeneity in a biologically meaningful way.

In the next section, we describe the multistage carcinogenesis model and introduce an extension by mixture. Then we will give a series of identifiability results for both the multistage model and some mixture models. Finally, we apply the model to human incidence data before giving some concluding remarks.

## Mathematical Model Formulation

### The Multistage Carcinogenesis Model

We will work with a simplified version of the multistage model, but one that is general enough to incorporate the two main features of the carcinogenesis process: the sequence of mutations and the clonal expansion. We make the following assumptions:

1. A cell must undergo *n *mutational events to get initiated.

2. The number of cells at risk, *N*_0_, is constant over time.

3. The number of newly generated initiated cells is a (non-homogeneous) Poisson process with intensity *λ*_*I*_(*t*).

4. An initiated cell gives rise to a clonal expansion according to a birth-and-death process with emigration, i.e. in a short time interval (*t*, *t *+ Δ*t*) an initiated cell divides in two initiated cells with rate *β*, dies or differentiates with rate *δ*(<*β*), and divides into one initiated and one malignant cell with rate *μ*.

5. Once a promoted cell is generated, its growth is deterministic, and we neglect the time needed to grow to detectable tumor size.

6. The system starts with all at risk cells in the normal state and the different cells act independently of one another.

**Figure 1:**
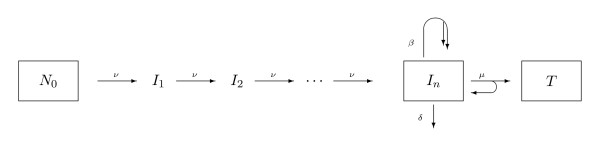
**The multistage carcinogenesis model**. *N*_0 _denotes the number of normal stem cells. To get initiated, a normal cell accumulates *n *consecutive mutations, where *ν *denotes the mutation rate per cell per year for the gene in question. The number of cells having *k *mutations is noted *I*_*k*_, 1 ≤ *k *≤ *n*. The fully initiated cells, *I*_*n*_, expand according to a birth-and-death process. These cells give rise to tumor cells *T *if a further event happens, and *μ*/(*μ *+ *β*) can be interpreted as the probability of such a malignant transformation during a cell division.

The model is shown schematically in Figure 1. Note that the above assumptions are standard in carcinogenesis modeling, and the possibility of generalization (for example to time dependent *N*_0_) has been discussed by several authors. However, we choose this simplified version in order to limit the complexity of our baseline model. Note also that for *n *= 1 we get the classical TSCE model.

A detailed discussion and derivation of the survivor and hazard functions of this multistage model can be found in [11,12] and [10]. These authors show that the survivor function for tumor onset can be represented as

(1) $$S(t;n)=\exp\left\{-\int_{0}^{t}\lambda_{I}(x)F_{P}(t-x)\text{d}x\right\}.$$

In this expression

(2) *λ*_*I*_(*x*) = *nν*^*n*^*N*_0_*x*^*n*-1 ^

is the intensity of initiation. The function *F*_*P *_(*x*) is the cdf for the waiting time for the first malignant transformation within a clone starting with one initiated cell at time 0. This cdf is improper since a clone of initiated cells dies out with a probability greater than 0 if *δ *> 0. Its exact form is

(3) $$F_{P}(x)=\frac{\left(\theta +\Delta )(\theta -\Delta )(e^{-\Delta x}-1\right)}{2\beta [(\theta +\Delta )e^{-\Delta x}-(\theta -\Delta )]},$$

where *θ *= *β *- *δ *- *μ *and $\Delta =\sqrt{{\left(\beta +\delta +\mu \right)}^{2}-4\beta \delta }$.

Researchers have expressed concern about the approximations used in carcinogenesis models. This issue was raised in a review paper by [13] and has inspired [11] and [14]. Our formula above is exact and uses the method based on integration cited by [[11], top of p. 1080]. The remaining simplifications in our model, in particular the constancy of *N*, are for convenience and do not affect the conclusions of the paper. As a general comment, it should be noted that the term two-stage refers to different things in different papers. It is, for example, possible to model clones via compartments or via branching processes. Both may employ the same parameter notation, but the interpretation will be quite different. Care has to be taken, if one wishes to stay close to biological reality. For further comments, see [[13], section 2.1].

The hazard function can be easily calculated from the survivor function, *h*(*t*; *n*) = *-*d log *S*(*t*; *n*)/d*t*. In order to deduce the asymptotic behavior of the hazard for several *n*, we note that *h*(*t*; *n*) can be written in terms of a recursion, *h*(*t*; 1) = *νN*_0_*F*_*P*_(*t*) and *h*(*t*; *n*) = $n\nu \int_{0}^{t}h(u;n-1)\text{d}u$ for *n *≥ 2. Therefore, when *n *= 1 the hazard levels off as *t *goes to infinity. More precisely, the hazard of the TSCE model goes to the finite asymptote

*νN*_0_·P(a clone of initiated cells does not die out).

But the hazard grows to infinity if *n *≥ 2. In both cases *h*(*t*; *n*) is strictly monotonic increasing with *t*. The unboundedness of the hazard is due to the simplifications in the model. This may lead to appreciable differences at low ages in some types of cancer or under hightened exposure. It is typically of lesser importance in human studies.

The monotonicity properties of hazard curves are not in agreement with observed incidence curves from human population data. Such data typically shows very low incidence up to about the age of fifty, a sudden and sharp increase between about the ages of fifty and eighty, and a subsequent leveling off and a decrease for the very old. This behavior at old ages is not captured by the hazard curves *h*(*t*; *n*). However, it can be modeled very easily by incorporating a frailty effect as we will show in the application later on.

### Extension of the Model by the Use of Mixture Distributions

An observed human population is heterogeneous. Though the process of cancer development is similar for everyone, parameters may vary between individuals. Since all parameters of the model are biologically meaningful, we aim at modelling heterogeneity directly through these parameters. We thus propose to consider some of the biological parameters – one at a time – as random variables.

Let *θ *be such a parameter and let *G*(*θ*) be a distribution function for *θ*. Then, we will denote by *S*(*t*|*θ*) the survivor function of the multistage model (1) for a given value *θ*, whereas the population survivor function is

(4) *S*(*t*) = ∫*S*(*t*|*θ*)dG(*θ*).

Under certain regularity conditions an analogous representation holds for the hazard function

$$h(t)=\int h(t|\theta )\frac{S(t|\theta )}{S(t)}\text{d}G(\theta ).$$

The distribution function *G *must then be selected based on the biological parameter *θ *chosen. If we consider for example *θ *= *n*, the number of mutations needed for initiation, it is natural to choose a finite distribution, i.e. P(*θ *= n_i_) = *π*_i _for a fixed set {*n*_1_,...,*n*_*g*_} ⊂ ℕ such that $\sum \pi_{i}=1$. This would correspond to *g *population subgroups having inherited different numbers of initiating mutations. The model could also be interpreted as a multiple pathway model, where *g *pathways involving different numbers of mutations can lead to cancer. Other interesting choices focus on promotion, for example *θ *= *β *- *δ*, the growth advantage of initiated cells, or *θ *= *μ*, the promotion rate. These two cases would be consistent with both a finite or a continuous distribution as long as its support is contained within biologically reasonable bounds.

## Identifiability

Before fitting our mixture model to observed incidence data, the identifyability issue has to be considered. The parameters of the TSCE model cannot be uniquely determined based on incidence data. Some of the papers relevant to this issue are [15,16], and [17].

### Identifiability of the Multistage Model

The parameters of our base model (Eq. 1, 2, 3) are *n*, the number of initiating mutations, and *ψ *= (*N*_0_, *ν*, *β*, *δ*, *μ*), sizes and rates. When fitting *S*(*t*|*n*, *ψ*), the survivor function of the multistage model given in (1), these six parameters cannot all be fitted separately. We will show that *n *and the follwing three combinations are, however, uniquely determined

$$\{\begin{array}{lll} p & = & \beta /(\nu^{n}N_{0}),\\ q & = & \delta -\beta +\mu ,\\ r & = & {\left(\beta +\delta +\mu \right)}^{2}-4\beta \delta . \end{array}$$

In order to deal with the discrepancy between the six parameters and the four identifyable features, we will hold the two parameters *N*_0 _and *δ *fixed (see also [18]). To determine *N*_0 _= *Number of stem cells in a given tissue *some preliminary biological estimate is needed. For the death rate we mainly focus on the choice *δ *= 0, which implies *γ *= *β *- *δ *= *β*, so that *β *simply describes the growth advantage of initiated cells.

#### The Number of Mutations for Initiation

Is *n *identifiable in the multistage model or could a change in *n *be compensated by some adjustment of the biological parameters *ψ *so that finally the same survivor function resulted? The answer to this question is no, because the behavior of *S*(*t*|*n*, *ψ*) at the origin is enough to determine *n*.

**Proposition 1. ***If for two parameter choices *(*n*, *ψ*), *and *($\tilde{n}$, $\tilde{\psi }$) *we have S*(*t*|*n*, *ψ*) ≡_*t *_*S*(*t*|$\tilde{n}$, $\tilde{\psi }$), *then n *= $\tilde{n}$.

*Proof: *Direct calculation shows that

$$\begin{array}{lll} S^{\left(k\right)}(0|n,\psi ) & = & \begin{array}{cc} 0 & \text{for }k=1,2,...,n,\text{ and} \end{array}\\ S^{\left(n+1\right)}(0|n,\psi ) & \neq & 0, \end{array}$$

for all *n *∈ {1, 2,...}, where *S*^(*k*) ^= *d*^*k*^*S*/*dt*^*k*^. The proposition is a direct consequence.

This result is mainly of theoretical interest and cannot be used to estimate *n*. In practice, for some tissues one can fix *n *according to the available biological theory. For example in colon cancer it is commonly assumed that two mutations are necessary for initiation, followed by a third one for malignant transformation (see [19]). In cases where no biological reasoning is available, we suggest to fit the model for several choices of *n*. The form of the intensity of initiation given in expression (2) shows that estimates for *ν *are highly sensitive to the choice of *n*. Results within biologically reasonable limits will thus be obtained only for very few values *n*.

#### Growth and Mutation Rates

For *n *= 1 it has been shown in [16] that three functions of *ψ *are uniquely determined by *S*(*t*|*n *= 1, *ψ*). Their proof can be generalized to *n *≥ 1. This is intuitively plausible. Given *n*, the intensity of initiation depends on the product *N*_0_*ν*^*n*^, but not on *N*_0 _and *ν *individually. And the speed at which a clone of initiated cells grows depends only on the difference *β *- *δ*, but not on the actual pair *β*, *δ*.

**Lemma 2. ***Let *(*n*, *ψ*) *and *($\tilde{n}$, $\tilde{\psi }$) *be two sets of parameters such that S*(*t*|*n*, *ψ*) ≡_*t *_*S*(*t*|$\tilde{n}$, $\tilde{\psi }$). *Then we have*

$$\nu^{n}N_{0}F_{P}(t;\psi )\equiv_{t}{\tilde{\nu }}^{n}{\tilde{N}}_{0}F_{P}(t;\tilde{\psi }).$$

*Proof: *Let us define the integral *I*(*t*; *n*, *ψ*) = $\int_{0}^{t}\lambda_{I}(t-x)F_{P}(x)\text{dx}$. This means that *S*(*t*|*n*, *ψ*) = exp{-*I*(*t*; *n*, *ψ*)}. Note that by Proposition 1 we have *n *= $\tilde{n}$. So we must show that if

(5) $$I(t;n,\psi )\equiv I(t;n,\tilde{\psi }),$$

then

$$\nu^{n}N_{0}F_{P}(t;\psi )\equiv {\tilde{\nu }}^{n}{\tilde{N}}_{0}F_{P}(t;\tilde{\psi }).$$

First, we transform *I*(*t*; *n*, *ψ*) via (*n *- 1) repeated integrations by parts into a *n*-fold integral. Next, we differentiate this expression *n *times with respect to *t*, to obtain

$$\frac{d^{n}}{dt^{n}}I(t;n,\psi )=n!\nu^{n}N_{0}F_{P}(t;\psi ).$$

Application of these two steps to both sides of (5) proves the result.

### Identifiability of the Mixture Structure

Besides the parameters of the multistage model itself, we must also investigate the identifiability of the newly introduced mixture structure. Let G be a family of distribution functions for a certain parameter *θ*. Then, G induces the family of mixture models

S={∫S(t|θ)dG(θ);G∈G}.

Family S is said to be identifiable with respect to G, if

$$\int S(t|\theta ){\text{dG}}_{1}(\theta )\equiv_{\text{t}}\int \text{S}(\text{t}|\theta ){\text{dG}}_{2}(\theta )\Rightarrow {\text{G}}_{1}\equiv_{\theta }{\text{G}}_{2}$$

holds for all *G*_1_, *G*_2 _∈ G. In other words, the population survivor function must uniquely determine the underlying mixing distribution within a pre-specified family.

This condition turns out to be hard to verify in general settings and we must focus on special cases. A very useful result was given in [20] for finite mixtures. Let Θ be a set of possible parameter values {*θ*_1_, *θ*_2_,...} such that *θ*_1 _<*θ*_2 _< ... . Then the finite mixture model $S(t)=\sum_{i=1}^{g}\pi_{i}S(t|\theta_{i})$ is identifiable if

(6) $$\exists a\in \mathbb{R}\cup \infty \text{ such that }\underset{t\to a}{\lim}\frac{S(t|\theta_{i+1})}{S(t|\theta_{i})}=0,\;\forall i.$$

This condition ensures identifiability of all finite mixtures of the survivor functions {*S*(*t*|*θ*_*i*_); *i *= 1, 2,...} even without specifying the number of components *g*. Teicher's result requires additional regularity conditions, but these are trivially satisfied in the case of the multistage model (1).

#### Initiation

The multistage model we consider here describes initiation as a sequence of discrete events, namely rate limiting mutations, which lead to a cell capable of accelerated growth. A biological mechanism generating heterogeneity at this stage are germ line mutations of the genes involved, leading to individuals starting life with all cells in an intermediate stage. Mathematically, this means that the population survivor function is

$$S(t)=\sum_{i=1}^{g}\pi_{i}S(t|n_{i},\psi ).$$

The next proposition shows that such a mixture is identifiable.

**Proposition 3. ***The family of finite mixture models induced by *{*S*(*t*|*n*, *ψ*); *n *= 1, 2,...} *is identifiable*.

*Proof: *We show that condition (6) is satisfied. The initiation incidence rates can be written recursively

$$\lambda_{I}(t;n+1)=\frac{n+1}{n}\nu t\lambda_{I}(t;n).$$

Thus, for $t>t_{1}:=\frac{2n}{\nu (n+1)}$,

$$\begin{array}{l} \begin{array}{l} \int_{0}^{t}\lambda_{I}(x;n)(\frac{n+1}{n}\nu x-1)F_{P}(t-x)\text{dx}\\ \geq \int_{0}^{t_{1}}\lambda_{I}(x;n)(\frac{n+1}{n}\nu x-1)F_{P}(t-x)\text{dx}+\underset{:=\Lambda (t)}{\underset{︸}{\int_{t_{1}}^{t}\lambda_{I}(x;n)F_{P}(t-x)\text{dx}}}. \end{array} \end{array}$$

Since *S*(*t*|*n*, *ψ*) → 0 for *t *→ ∞, we have Λ (*t*) → ∞ for *t *→ ∞. This implies

$$\frac{S(t|n+1,\psi )}{S(t|n,\psi )}\;\overset{t\to \infty }{\to }0.$$

#### Promotion

Promotion is a complicated process and both genetic and epigenetic factors seem to be involved. Therefore, heterogeneity can be due to many different mechanisms. In the context of the multistage model, there are two main parameters these agents can influence: the growth advantage of initiated cells, *γ*, and the rate of malignant transformation, *μ*.

We can derive a result similar to the one in the previous section. Let there be a discrete set of *γ*-values 0 <*γ*_1 _<*γ*_2 _< ... . Note that we consider *δ *= *γ*_*i *_- *β*_*i *_as fixed, i.e. we assume in fact that there is an analogous sequence of *β*_*i*_. From now on, we will write *ψ *for the parameter vector (*n*, *N*_0_, *ν*, *δ*, *μ*).

**Proposition 4. ***The family of finite mixture models induced by *{*S*(*t*|*γ*_*i*_, *ψ*); *i *= 1, 2,...} *is identifiable*.

*Proof: *We will first check condition (6) in the case *δ *> 0. We have

$$\frac{S(t|\gamma_{i+1},\psi )}{S(t|\gamma_{i},\psi )}=e^{-\int_{0}^{t}\lambda_{I}(t-x)[F_{P}(x|\gamma_{i+1})-F_{P}(x|\gamma_{i})]\text{dx}},$$

and the assumption *δ *> 0 implies that *F*_*P *_is improper and converges to a limit *a*(*γ*, *δ*) < 1 as t → ∞. The value 1 - *a*(*γ*, *δ*) is the probability that a clone of initiated cells (generated by a single initiated cell at time *t *= 0) eventually dies out without ever giving rise to a promoted cell. The assumption *γ*_*i*+1 _> *γ*_*i *_implies that *a*(*γ*_*i*+1_,*δ*) > *a*(*γ*_*i*_, *δ*), and as a consequence

$$\int_{0}^{t}\lambda_{I}(t-x)[F_{P}(x|\gamma_{i+1})-F_{P}(x|\gamma_{i})]dx\overset{t\to \infty }{\to }\infty .$$

Let us next consider the case *δ *= 0, and thus *γ*_*i *_= *β*_*i*_. The function *F*_*P *_is in this case equal to

$$F_{P}(x|\beta )=\frac{\mu -\mu e^{-(\beta +\mu )x}}{\mu +\beta e^{-(\beta +\mu )x}}.$$

Using the mean value theorem we have

$$F_{P}(x|\beta_{i+1})-F_{P}(x|\beta_{i})=(\beta_{i+1}-\beta_{i}){\frac{\partial }{\partial \beta }F_{P}(x|\beta )|}_{\tilde{\beta }},$$

where $\tilde{\beta }$ lies between *β*_*i *_and *β*_*i*+1_. A direct calculation shows that

1. $\frac{\partial }{\partial \beta }F_{P}(0|\tilde{\beta })=0$ = 0,

2. $\frac{\partial }{\partial \beta }F_{P}(x|\tilde{\beta })$ is non-negative for all *x *≥ 0, and

3. $\frac{\partial }{\partial \beta }F_{P}(x|\tilde{\beta })$ asymptotically goes to 0 as *x *→ ∞.

Let *t*_0 _be the (unique) maximum of $\frac{\partial }{\partial \beta }F_{P}(x|\tilde{\beta })$. It follows that

$$\begin{array}{l} \int_{0}^{t}\lambda_{I}(t-x)\frac{\partial }{\partial \beta }F_{P}(x|\tilde{\beta })\text{dx}\\ =\underset{>\lambda_{I}(t-t_{0})\int_{0}^{t_{0}}\frac{\partial }{\partial \beta }F_{P}(x|\tilde{\beta })\text{dx}}{\underset{︸}{\int_{0}^{t_{0}}\lambda_{I}(t-x)\frac{\partial }{\partial \beta }F_{P}(x|\tilde{\beta })\text{dx}}}+\underset{>0}{\underset{︸}{\int_{t_{0}}^{t}\lambda_{I}(t-x)\frac{\partial }{\partial \beta }F_{P}(x|\tilde{\beta })\text{dx}}}. \end{array}$$

This shows that

$$(\beta_{i+1}-\beta_{i})\int_{0}^{t}\lambda_{I}(t-x)\frac{\partial }{\partial \beta }F_{P}(x|\tilde{\beta })\text{dx}\overset{t\to \infty }{\to }\infty ,$$

which completes the proof.

The same idea could be applied to parameter *μ*. Though the biological interpretation of such a frailty model would be different, technically no new issues arise, and similar results can be established.

## Fitting Mixture Models

We will now apply the proposed mixture model to the human lung cancer incidence data from [10]. These authors have studied mortalities due to lung cancer in different birth cohorts of European Americans, namely those born in the 1880s, 1890s, 1900s and 1920s. The data comes in form of a vector

(*r*_*i*_, *o*_*i*_), *i *= 1,...,*N*,

where *r*_*i *_counts the population at risk and *o*_*i *_counts the observed cancer cases during the time interval [*t*_*i*_, *t*_*i*+1_). The data is discussed in [21] and is publically available ([22]). Additional information is given in [23].

**Figure 2:**
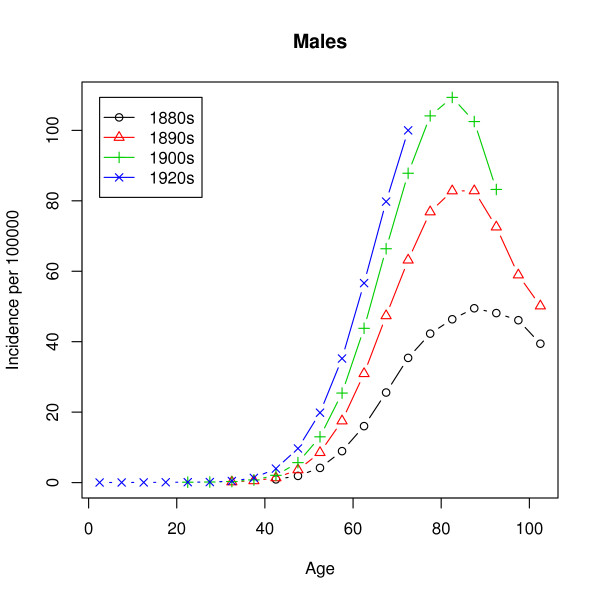
**Observed incidence (males)**. Observed lung cancer incidence rates in the United States for four birth cohorts. The population considered are the males of European descent.

**Figure 3:**
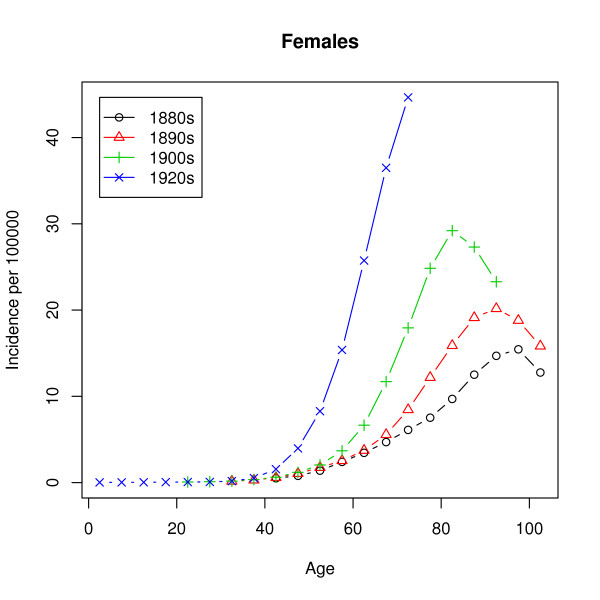
**Observed incidence (females)**. Observed lung cancer incidence rates in the United States for four birth cohorts. The population considered are the females of European descent.

In our case, the data is grouped into 5-year age groups: 0–4 years, 5–9 years, 10–14 years and so on. Figures 2 and 3 show the raw hazard estimates

$${\hat{\lambda }}_{i}=\frac{o_{i}}{r_{i}(t_{i+1}-t_{i})}.$$

As mentioned earlier, the observed hazard has a peak at around 80 years and a decrease for the higher ages, while the hazard of the multistage model given by (1) is strictly monotonic increasing. Estimation of the parameters by analytic graduation as described later on leads to extremely poor fits, which for all ages above 30 and for all birth cohorts give quite useless predictions. The fault does, however, not lie with the methods of estimation but rather with the model. Thus, using the inverse of the variance of the estimated incidence rates as weights leads to almost the same poor fit. The unmixed multistage model does not succeed in describing the incidence rates in Figures 2 and 3. We will come back to this failure.

Two component mixtures on the other hand are flexible enough to provide good fits. We will illustrate this using the *γ*-frailty model

(7) *S*(*t*) = *π*_*l*_*S*(*t*|*γ*_*l*_) + *π*_*u*_*S*(*t*|*γ*_*u*_),

where 0 ≤ *π*_*l *_≤ 1, *π*_*l *_+ *π*_*u *_= 1, 0 <*γ*_*l *_<*γ*_*u*_. To get identifiability, we will fix *N*_0 _and *δ*. But in order to get stable estimates, we fix also *γ*_*l *_and *n*. Note that the parameters we estimate have a restricted domain of definition,

(*π*_*l*_, *ν*, *γ*_*u*_, *μ*) ∈ (0,1) × ℝ_+ _× (*γ*_*l*_, ∞) × ℝ_+_.

We will use suitable transformations to respect these constraints.

### Maximum Likelihood Estimation

We treat the failures from competing causes as right censorings. This means for each time interval [*t*_*i*_, *t*_*i*+1_) we observe *o*_*i *_failures due to cancer and have *c*_*i *_= *r*_*i *_- *r*_*i*+1 _- *o*_*i *_censored individuals. Under the assumption of independent and uninformative censoring, the likelihood function *L*(*π*_*l*_, *ν*, *γ*_*u*_, *μ*|*N*_0_, *δ*, *n*, *γ*_*l*_) is given by

$$\prod_{i=0}^{N}{\left(S(t_{i})-S(t_{i+1})\right)}^{o_{i}}S{\left(t_{i}\right)}^{c_{i}}.$$

**Figure 4:**
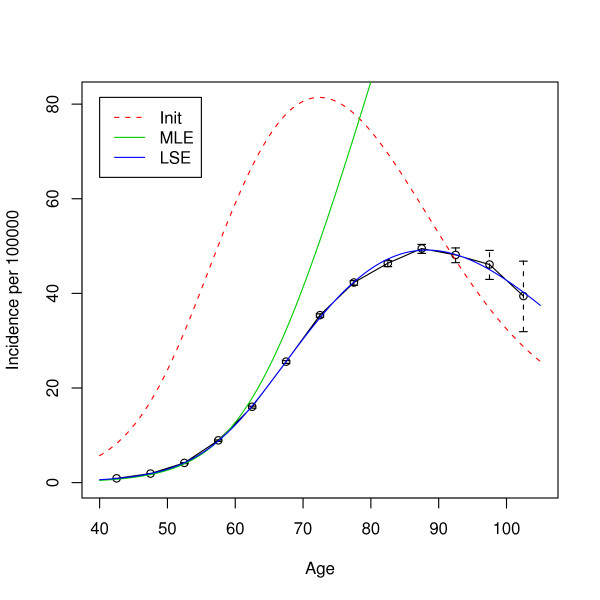
**ML and LS fits**. Lung cancer incidence rates for the European American males born in the 1880s. The superposed curves show the fitted hazards of the carcinogenesis model (7) based on the MLE and least squares. In the fitting process, *N*_0 _= 10^10^, *δ *= 0, *n *= 2 *γ*_*l *_= 10^-4 ^were kept constant. The initial value for the remaining parameters were *π*_*l *_= 0.97, *γ*_*u *_- *γ*_*l *_= 0.2, *ν *= 10^-6.5 ^and *μ *= 10^-5^.

By numerically optimizing this likelihood, we observe a strange behavior of the MLE. Figure 4 shows the data from the males 1880s cohort along with the models corresponding to the MLE, the least squares fit LSE, and the starting value of the numerical optimization. As we can see, the MLE fails completely to catch the behavior of the observed incidence at old ages; only the first few data points are well fitted. Convergence to this model seems even more astonishing when we consider the initial model. The chosen starting value is far away from the data in terms of fit, but it is close to the observed hazard in terms of shape. Furthermore, the model corresponding to the LSE fits the observed hazard very closely. This shows that the parametric family we apply to the data does indeed contain models that can fit. But in this example, likelihood and fit do not measure the same thing. The huge discrepancy, however, is intriguing. The strange behavior of the MLE is caused by several effects. One aspect is model mis-specification in relation with the special metric used in likelihood based inference. The data is not really generated by our multistage model, while the MLE corresponds to the survivor function that minimizes the Kullback-Leibler distance to the observed empirical survivor function. But this is a very special metric and can produce obviously strange results in some cases.

**Figure 5:**
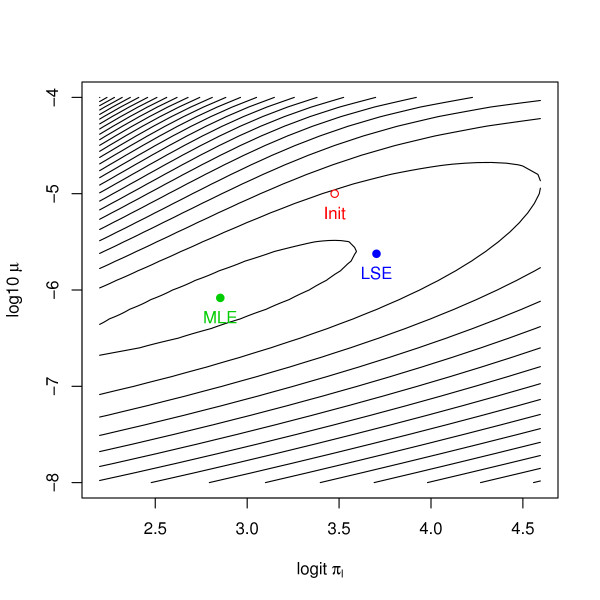
**Log-likelihood surface**. Contour plot of the log-likelihood surface. The parameter space is reduced by keeping all the parameters except two constant. The two parameters shown are logit*π*_*l *_and log_10 _*μ*.

In mechanistic modeling, likelihood based inference is often difficult due to local maxima and/or low curvature around the maxima. Both problems apply to our case. Our likelihood-surface is multimodal because the different biological parameters compete. This problem can be avoided by extensive use of the available biological knowledge. If we have good starting values and restrict attention to biologically reasonable intervals, then the likelihood surface is unimodal in that domain. The second problem is more difficult to treat. Even for identifiable parameters the likelihood surface is often extremely flat around its maximum. Figure 5 gives the contour plot of the log-likelihood for a reduced parameter space. That is, we take model (7), but fix all parameters except *ψ *= (logit*π*_*l*_, log_10 _*μ*). The log-likelihood essentially has a ridge starting the upper-right corner and running downwards as one moves to the left. This means that only a combination of the two parameters can be estimated precisely, but not both separately. The log-likelihood values of the estimates in Figure 5 are *l*(${\hat{\psi }}_{\text{ML}}$) = -1.338·10^6 ^and *l*(${\hat{\psi }}_{\text{LS}}$) = -1.355·10^6^. While these values appear to be close, they are in fact quite different in the likelihood metric, because

$$2(l({\hat{\psi }}_{\text{ML}})-l({\hat{\psi }}_{\text{LS}}))≃32600\gg q\chi_{2}^{2}(0.999)\approx 13.8.$$

**Figure 6:**
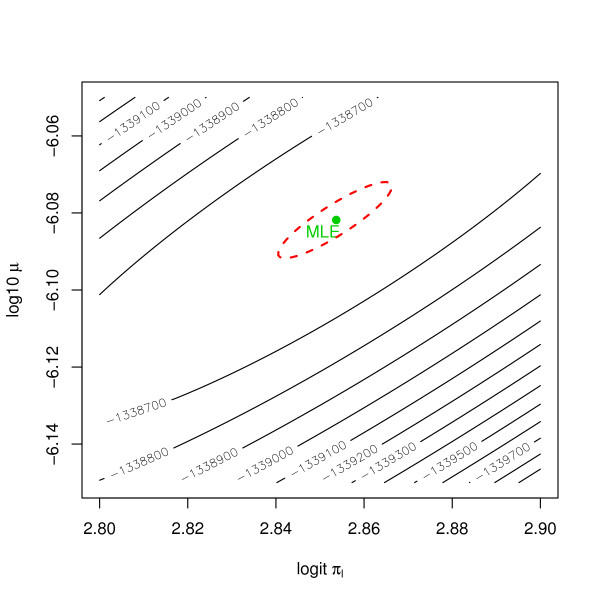
**Log-likelihood surface (zoomed)**. Contour plot of the log-likelihood surface as shown in Figure 5. The plot zooms in on the MLE and in addition contains a 95% confidence ellipsoid.

A 95% confidence region determined by a likelihood-ratio test is shown in Figure 6. Note how small this confidence set is. So, not only does the likelihood technology give badly fitting hasard rates, it is also overly optimistic about having found the right values.

The most important reason that leads to the failure of the MLE in our application, however, is the heavy censoring. We deal with human cancer incidence data. This means we consider a rare event, and most members of the population fail from competing causes. In the data set we are considering there are tens of millions individuals at risk at the first time points, but only some tens of thousands at the last one. In order to illustrate the impact of censoring, we will construct a sequence of artificial data sets that lead to the same raw hazard estimates, but differ in the degree of censoring. As before, we note by (*r*_*i*_, *o*_*i*_) the real data set. Let us define the points (${\tilde{r}}_{i}^{k}$, ${\tilde{o}}_{i}^{k}$) by

(8) $$\begin{array}{ccc} {\tilde{r}}_{i}^{k}=10^{6}-i\cdot k10^{4}, & \text{and} & \frac{{\tilde{o}}_{i}^{k}}{{\tilde{r}}_{i}^{k}}=\frac{o_{i}}{r_{i}}. \end{array}$$

**Figure 7:**
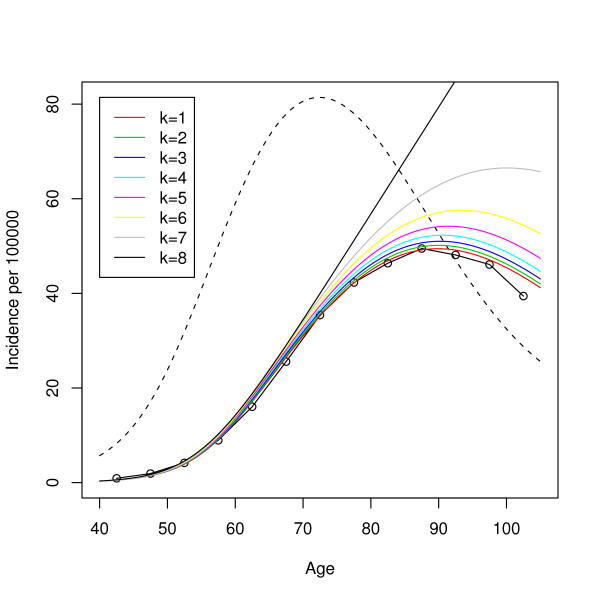
**ML fits to artificial data**. The hazard curves corresponding to the maximum likelihood fits for the data sets constructed according to (8) for different values of *k*.

**Figure 8:**
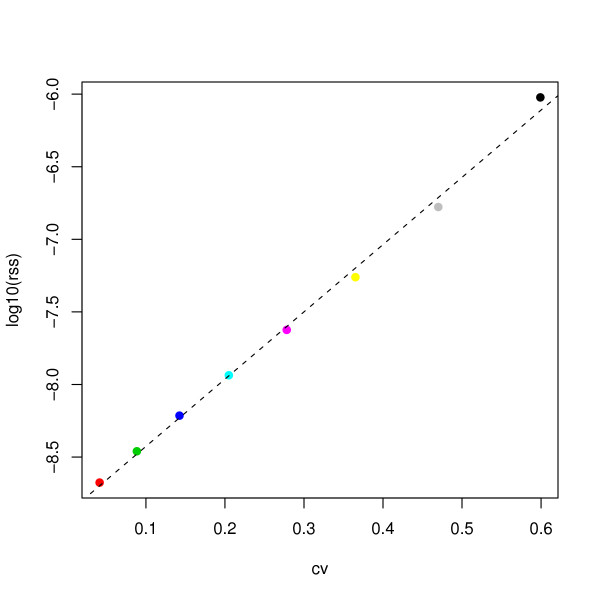
**RSS of ML fits**. The logarithm of the residual sums of squares of the various fits shown in Figure 7 as a function of the coefficient of variation of the size of the at risk set. Note that for the real data set we have cv(*r*_0_,...,*r*_12_) ≈ 0.77.

That is we start with a population of size 10^6 ^and suppose that during every time interval exactly *k*10^4 ^individuals die – either due to cancer or due to competing causes. We then fit model (7) by maximum likelihood as before (we consider again the four parameters *π*_*l*_, *ν*, *γ*_*u*_, *μ *as unknown). Figure 7 gives the estimated models for *k *= 1,...,8. The MLE behaves better for small *k *than for large *k*. In Figure 8 we calculated the residual sums of squares (RSS) for these models, which seem to increase exponentially with the coefficient of variation of the ${\tilde{r}}_{i}^{k}$ sequence,

$${\text{cv}}_{k}=\frac{\text{sd}({\tilde{r}}_{0}^{k},\ldots ,{\tilde{r}}_{12}^{k})}{\text{mean}({\tilde{r}}_{0}^{k},\ldots ,{\tilde{r}}_{12}^{k})}.$$

The above example shows that the MLE is dominated by the points corresponding to large "at risk" sets. The LSE, on the other hand, works fine, since it attributes equal weight to all age intervals. This makes one wonder whether a weighted least squares approach would suffer from the same problem as the MLE. If we give for example weights proportional to the population at risk, would the LSE break down as well? The answer to this question is clearly no. Considering Figure 4 once again, we realize that there is a model that fits all the data points very accurately. This model will be good even if we downweight the contribution to the RSS of the points at high ages. Any weighted least squares approach will select a model that is very close to the standard LSE.

### Analytic Graduation

The LS estimates shown in the previous figures were obtained by analytic graduation, which is a standard procedure to fit continuous curves to discretized data. A detailed discussion of the procedure and derivation of asymptotic results can be found in [24,25].

**Figure 9:**
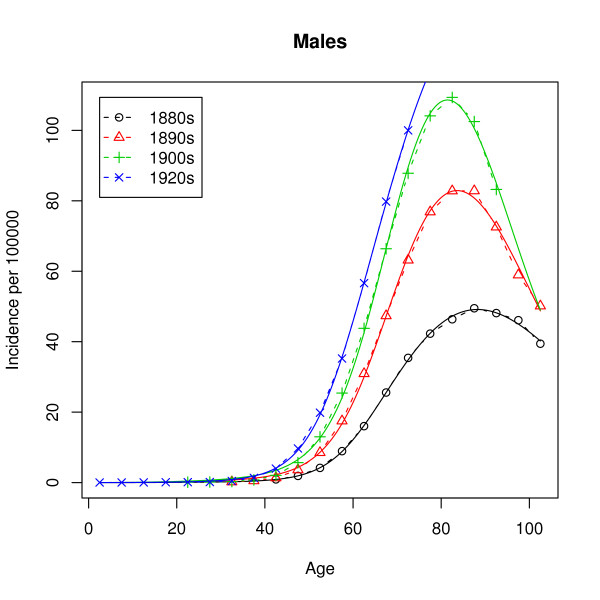
**LS fits (males)**. Observed (dashed lines) and modeled (solid lines) incidence rates for the data from Figure 2.

**Figure 10:**
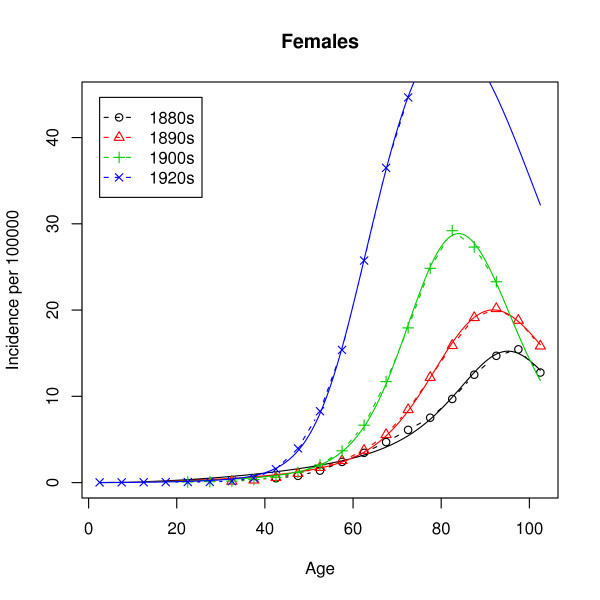
**LS fits (females)**. Observed (dashed lines) and modeled (solid lines) incidence rates for the data from Figure 3.

Figures 9 and 10 show model (7) fitted to the different cohorts. The model successfully reproduces the observed data. Table 1 gives the corresponding parameter estimates. Note that these values are conditional given *N*_0_, *δ*, *γ*_*l *_and *n*. The value *N*_0 _acts as a scale parameter. Changes in *N*_0 _are compensated by *ν *such that the product *N*_0 _*ν*^*n *^stays more or less constant. The other parameters also remain quite stable. The effect of *δ *is rather fuzzy, no clear conclusions emerge. In all cases where enough data at old ages is available (i.e. all but the 1920s cohort), the estimated proportion of the population at high risk, ${\hat{\pi }}_{u}$, is not sensitive to changes in the fixed parameter values. The peak of the observed hazard determines ${\hat{\pi }}_{u}$ quite accurately. Finally, reasonable results can also be obtained for *n *= 3, while other choices of *n *produce unrealistic estimates for at least some of the parameters. Note that good fits can be achieved only as long as *γ*_*l *_is small enough. We set *γ*_*l *_= 10^-4 ^in the models given here.

**Table 1:** Conditional parameter estimates given the fixed values *n *= 2, *N*_0 _= 10^10^, *δ *= 0 and *γ*_*l *_= 10^-4^.

Cohort	Males				Females			
	${\hat{\pi }}_{u}$	$\hat{\nu }$	${\hat{\gamma }}_{u}$	$\hat{\mu }$	${\hat{\pi }}_{u}$	$\hat{\nu }$	${\hat{\gamma }}_{u}$	$\hat{\mu }$
1880s	0.021	2.5 × 10^-7^	0.183	3.5 × 10^-6^	0.003	4.9 × 10^-7^	0.134	2.2 × 10^-6^
1890s	0.029	3.2 × 10^-7^	0.173	4.4 × 10^-6^	0.005	4.3 × 10^-7^	0.146	2.1 × 10^-6^
1900s	0.034	3.6 × 10^-7^	0.167	5.2 × 10^-6^	0.007	4.8 × 10^-7^	0.168	1.3 × 10^-6^
1920s	0.077	1.9 × 10^-7^	0.189	7.5 × 10^-6^	0.023	2.4 × 10^-7^	0.203	3.2 × 10^-6^

The least squares estimates for a single unmixed multistage model in which all the parameters except *N *and *δ *are fitted, lead to curves ressembling the maximum likelihood fit in Figure 3. The estimated parameters show that the simple multistage model attempts to distinguish between the earlier and later cohorts to a large extent by increasing the initiation rate *ν*. The increase is three-fold between 1880 and 1920 for the females and even six-fold for the males. The other parameters remain more or less constant across the cohorts. The incidence rates in females is much lower than in males. While the mixture model adjusts to this through an adjustment of the mixture weights, the single multistage model explains it by very different estimates of the promotion rate *μ *between the sexes.

**Figure 11:**
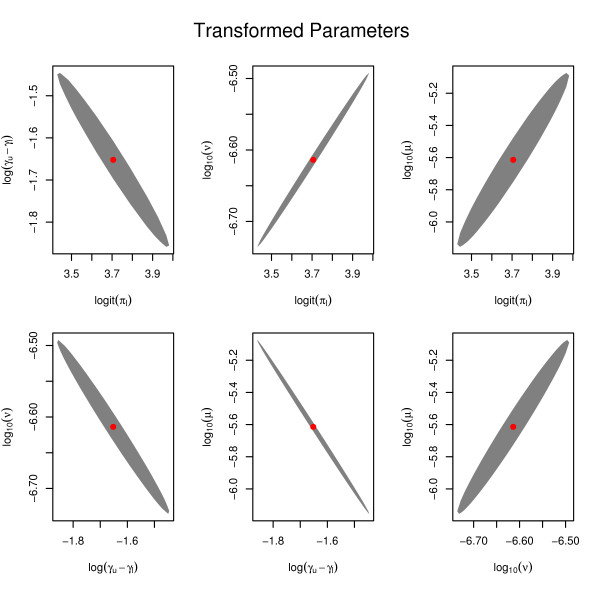
**95% asymptotic confidence region**. Projections of an asymptotic 95% confidence region (CR) for the parameters of the carcinogenesis model fit the the 1880s birth cohort of European American males. Note that we used the parametrization (logit(*π*_*l*_), log(*γ*_*u *_- *γ*_*l*_), log_10 _*ν*, log_10 _*μ*) in the fitting process.

In order to assess the accuracy of the given estimates, we use projections of a joint confidence region rather than marginal confidence intervals. In other words, we determine a confidence region *C *⊂ ℝ^4^, and then we look at projections of *C *on the six parameter plains spanned by the four parameter axis. The confidence region we get for the EAMs 1880s cohort is shown in Figure 11. The confidence region reveals the strong dependencies between the different parameters. Though a parametrization with such dependant parameters is unsatisfactory from a mathematical point of view, the dependencies might be interesting in biological terms. Not the two mutation rates *ν *and *μ *seem to compete, but rather the net growth rate *γ *and the two mutation rates. So at the extremes of the confidence region, we have models with high mutation rates but low proliferation of initiated cells, or models with low mutation rates but large cell growth. Note that the corresponding hazard curves are markedly different.

## Discussion

The *γ*-frailty model we fitted to our data is such that only one parameter involved in promotion is allowed to vary between population subgroups. Such a model would suggest a process where initiated cells are created in all individuals according to the same dynamics, but only in a small subgroup of the population promotion and malignant transformation happen. This is consistent with the fact that promotion depends on stimuli that might be present only in a fraction of the population. The proportion of high risk individuals, estimated by ${\hat{\pi }}_{u}$, reflects the change of the hazard curves between the 1880s and the 1920s cohorts. The sharp increase of maximal incidence in a relatively short period of time must be due to environmental factors such as occupational exposure and smoking.

We got satisfactory fits only when we allowed for two clearly separated population subgroups with a low risk group that runs a risk close to zero. This is consistent with the results reported by the other research groups that introduced frailty into carcinogenesis modeling. In [10] the estimated fraction at risk is very low. And also in [8] the estimated proportion of susceptibles is lower than 0.5%, though these authors worked with Scandinavian data on testicular cancer.

Besides the *γ*-frailty model we considered here, one could clearly build mixture models using other parameters. In particular the number of mutations for initiation, *n*, the rate of malignant transformation, *μ*, or the initiating mutation rate *ν *would be natural choices. The increase in lung cancer rates that was observed during the 20^th^century coincides with an increase in the fraction of smokers. Smoking might very well influence *μ *and *ν*. However, when applying such models to our data, no new issues arise, and we omit a detailed discussion here. Still, one needs to realize that all the mentioned models can fit the data equally well. This is not surprising, since we fit a relatively complex model to a very simple data structure. However, in all approaches we tested, the data suggested two component mixtures with a small high risk group and a large quasi immune group.

## Conclusion

We have studied an extension of the multistage carcinogenesis model by mixture. This allowed us to introduce population heterogeneity. The multistage model is a mechanistic model and all its parameters have a biological interpretation. Therefore, it is natural to introduce the notion of frailty in a biologically meaningful way. Such an approach is given by our mixture models, which can reproduce observed human lung cancer incidence data very accurately. Very good fits are achieved with very simple, two component mixtures also in cases where a continuous distribution might seem more adequate. However, the peak observed in the population hazard rates can be reproduced by continuous mixture models only when the population is clearly separated into a high risk and a low risk group. In other terms, the density of such a distribution would typically be bathtub shaped, closely resembling two component mixtures. Biological systems are often buffered. Small changes in the environment have no significant effect, and only after passing over some threshold value may abrupt changes in the system occur. Since we consider a late end-point, namely cancer, in a very complicated system, it is not surprising that we obtain in Section 4 mixture models that reflect such a buffering. It would be interesting to link the model with concrete biological mechanisms that are able to explain flip-flop processes. This would be an approach to understand how heterogeneity acts upon human carcinogenesis.

## Competing interests

The authors declare that they have no competing interests.
